# Biological and molecular characterization of classical swine fever challenge virus from India

**DOI:** 10.14202/vetworld.2015.330-335

**Published:** 2015-03-16

**Authors:** Parveen Kumar, Vikramaditya Upmanyu, Pronab Dhar

**Affiliations:** Division of Biological Standardization, Indian Veterinary Research Institute, Izatnagar, Uttar Pradesh, India

**Keywords:** Classical Swine fever, Challenge virus, molecular characterization, biological characterization, phylogeny

## Abstract

**Aim:**

The aim of this study was biological and molecular characterization of classical swine fever (CSF) challenge virus from India.

**Materials and Methods:**

CSF challenge virus maintained at Division of Biological standardization was experimentally infected to two seronegative piglets. The biological characterization was done by clinical sign and symptoms along with postmortem findings. For molecular characterization 5’-nontranslated region, E2 and NS5B regions were amplified by reverse transcription polymerase chain reaction and sequenced. The sequences were compared with that of reference strains and the local field isolates to establish a phylogenetic relation.

**Results:**

The virus produced symptoms of acute disease in the piglets with typical post-mortem lesions. Phylogenetic analysis of the three regions showed that the current Indian CSF Challenge virus is having maximum similarity with the BresciaX strain (USA) and Madhya Pradesh isolate (India) and is belonging to subgroup 1.2 under Group 1.

**Conclusion:**

Based on biological and molecular characterization of CSF challenge virus from India is described as a highly virulent virus belonging to subgroup 1.2 under Group 1 along with some field isolates from India and Brescia strain.

## Introduction

Classical swine fever (CSF) is most important contagious viral disease of pigs and wild boar and causes high mortality [1]. The disease is characterized by anorexia, lethargy, high fever, marked leukopenia, conjunctivitis, enlarged and discolored lymph nodes, respiratory signs and diarrhea, followed by death. Neurological signs are frequently seen, such as a staggering gait with weakness of hind legs, incoordination of movement, and convulsions. The CSF virus (CSFV) is able to cross the placenta of pregnant animals, thereby infecting fetuses during all stages of pregnancy [2,3]. Disease is controlled by vaccination of pigs with live attenuated swine fever vaccine or stamping out policy [4,5]. In India, pigs are vaccinated with lapinized swine fever vaccine and effectiveness of such vaccine is an important need for successful control of disease in the country. The efficacy of this vaccine is tested by the challenge of vaccinated and control pigs by virulent swine fever virus (Indian Pharmacopoeia, 2014). Thus, a well characterized swine fever virus both at the molecular and biological level is an essential requirement for efficacy testing of lapinized vaccine.

The genome of CSFV is a single strand RNA of the positive sense, approximately 12,300 nucleotides in length. It has non-translated regions at either end (5’NTR and 3’NTR) of its genome, encompassing a single open reading frame encoding a large protein that is cleaved into smaller fragments. These cleaved proteins include a N-terminal protease, four structural proteins (C, Erns, E1, E2) toward the 5’ end of the genome and seven non-structural proteins (NS1, NS2, NS3, NS4A, NS4B, NS5A, NS5B) in the 3’ two-thirds of the genome [6]. Three regions of the genome, i.e. 5’ UTR (150 bp), E2 envelope glycoprotein gene (190 bp) and NS5B polymerase gene (449 bp) [7] have most commonly been used for classifying CSF virulent and vaccine virus strains/isolates into 3 groups and 11 subgroups [8-10]. Group 1 includes most historical isolates, which are both highly virulent (used as challenge viruses) and vaccine viruses, while Group 2 and 3 includes current isolates causing epidemics in various countries [8]. In India, majority of outbreaks have reported from Group 1 isolates while few reports of 2.1 and 2.2 outbreaks have been increasingly reported [11].

In the present study, 5`NTR and NS5B region for typing and molecular characterization of the CSF challenge virus, while E2 region is used for molecular characterization. For biological characterization, two seronegative piglets were experimentally infected with CSF challenge virus.

The aim of the present study was biological and molecular characterization of CSF challenge virus from India.

## Materials and Methods

### Ethical approval

Animal experiments were approved by Committee for the Purpose of Control and Supervision of Experiments on Animals, Ministry of Environment and Forests, New Delhi and bio-security measures were taken during the whole study.

### Experimental infection and pathogenicity studies

The CSFV challenge virus, used in this study was kindly provided by Veterinary Biological Product Institute, Mhow, M.P., in 2005 and the same has been maintained in the Division of Biological Standardization by pig passages every year. The virus is being used for potency testing of CSF vaccine and also supplied to various State Biological Units.

In the present experiment, two seronegative piglets, aged around 2 months were used. Piglets were monitored for a period of 2 weeks prior to experiments for general clinical sign and rectal temperature. For the experimental infection, both piglets were injected subcutaneously into neck region with 10 ml 20% spleen suspension, containing challenge virus. Piglets were examined twice a day for the clinical signs and change in rectal temperature. Blood was collected (heparinized) at the peak rectal temperature for isolation of viral RNA. Total blood leukocyte count was calculated using Newberg’s hemocytometer using the standard method before infection and 11^th^ day post infection (dpi). The piglets were sacrificed after 11^th^ days for post mortem studies after moribund condition.

### Viral RNA isolation and polymerase chain reaction (PCR)

Total RNAs were extracted from the CSFV infected blood or spleen by TRIzol reagent (Life Technologies) as per the manufacturer’s protocol. The total RNAs were reverse transcribed to generate cDNA by MMLV-Reverse Transcriptase enzyme (Promega) using random hexamers. The cDNA obtained was further subjected to PCR for the three regions of 5’ NTR, E2 and NS5B using primers shown in Table-1.

**Table-1:** Primers used for amplification of 5`NTR, NS5B and E2 regions.

Region	Forward primer	Reverse primer	Annealing temperature (Ta)	Product size (bp)	References
5’NTR	5’ ATG CCC TTA GTA GGA CTA GCA 3’ (100–120)	5’ TCA ACT CCA TGT GCC ATG TAC 3’ (363-383)	50°C	284	[12]
NS5B	5’ GAC ACT AGY GCA GGC AAY AG 3’ (11138-11157)	5’ AGT GGG TTC CAG GAR TAC AT 3’ (11586-11567)	55°C	449	[7]
E2	5’ ATA TAT GCT CAA GGG CGA GT 3’ (3388-3407)	5’-ACA GCA GTA GTA TCC ATT TCT TTA-3’ (3695-3672)	56°C	308	[13]

5’ NTR=5’ non-translated regions

PCR reactions were performed in 25 μl of reaction mixture, which contained 2.5 μl of ×10 buffer, 200 μM of each dNTP, 10 pmol of each primer, 50 ng of each DNA sample, and 1.5 units of Taq polymerase (Thermo scientific). Amplification was carried out with initial denaturation step of 94°C for 4 min, followed by 35 cycles of 94°C for 1 min, annealing temperature (Ta) for 1 min, 72°C for 1 min, and a final extension of 72°C for 10 min. Annealing temperature (Ta) was standardized at 50°C, 55°C and 56°C for PCR of 5’ NTR, E2 and NS5B regions respectively (Table-1). The PCR products were analyzed with 1% agarose gel electrophoresis. The size of the amplified product was compared with the DNA ladder of 100 bp size.

### Sequencing and phylogenetic analysis

The PCR products were gel purified using QIAquick Gel Extraction Kit (Qiagen Inc. Valencia, CA, USA) as per the manufacturer’s protocol. The purified PCR products were sequenced using Sanger`s sequencing method and the partial genomic sequences of 5’ NTR, E2 and NS5B gene of Indian CSF challenge virus were submitted to GenBank and their accession numbers are JF903852 (5`NTR), JF903851 (E2), and JF903853 (NS5B).

Sequences for the phylogenetic analyses were obtained from already published [9,10,14,15] and derived from whole genome or partial genome data available in GenBank. For phylogenetic analysis and generation of the phylogenetic tree, a standardized protocol as described by Lowings *et al*. (1996) and Paton *et al*. (2000) was used. Phylogenetic analysis was performed for the 150 bp fragment for 5’ NTR, 308 bp fragment for E2, and 409 bp sequence for NS5B region of the CSF viral genome. The sequences from different sources were obtained and manually edited to obtain the desired sequence length for uniform alignment.

Alignment of nucleotides sequences were analyzed by the Clustal W method. Sequences were assembled into multiple sequences alignment. The phylogenetic tree derived from sequences was constructed by neighbor-joining method of MEGA version 6 [16]. To access the statistical reliability of the phylogenetic trees bootstrap values were generated at a repetition of 1000 times. For classification of the virus and grouping nomenclature as defined by Lowings *et al*. (1996) and Paton *et al*. (2000) were used as the starting point. Phylogenetic and molecular evolutionary analyses were conducted using MEGA version 6 [16].

## Results and Discussion

### Biological characterization

**Figure-1:**
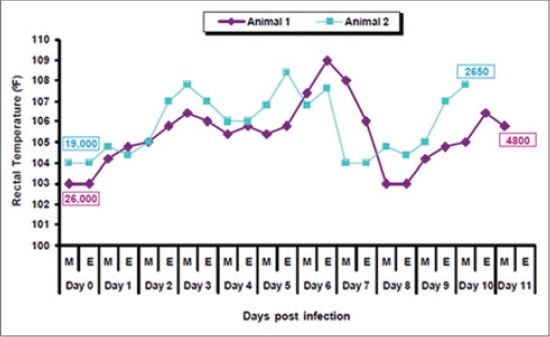
Thermal reaction in pigs following experimental infection with classical swine fever challenge virus

Both infected piglets showed typical clinical signs and thermal reaction post infection. Rise in body temperature was observed from 0 dpi to 6 dpi from 103°F to 109°F and 104°F to 108.4°F, after that temperature dropped down slowly (Figure-1). Blood was collected at peak of rectal temperature (6 dpi), which was used for molecular characterization studies. Total leukocyte count comparison showed extreme leucopenia on 10 dpi as compared to 0 dpi (26000 cells/mm^3^-4800 cells/mm^3^ and 19000 cells/mm^3^-2650 cells/mm^3^) (Figure-1).

**Figure-2:**
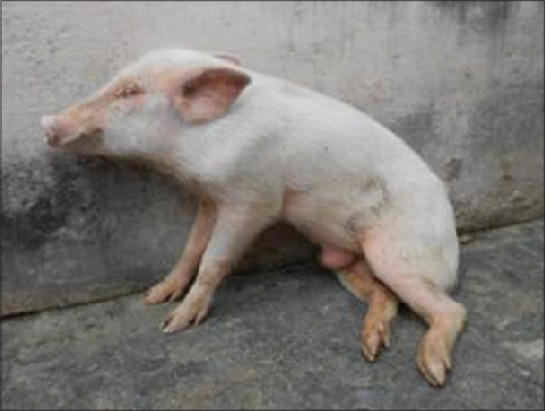
Posterior paresis in classical swine fever virus infected pig after 8 dpi.

From 3^rd^ to 4^th^ days onwards, animal had reduced feed intake, and after 4 dpi, animals were completely fed-off. After 5 days, dpi animals became recumbent with vigorous belly movement. After 6-8 dpi animals were recumbent, with dyspnea, eyes closed and inability to stand, and nervous signs were observed. Posterior paralysis was observed in both the animals 8 dpi (Figure-2).

**Figure-3:**
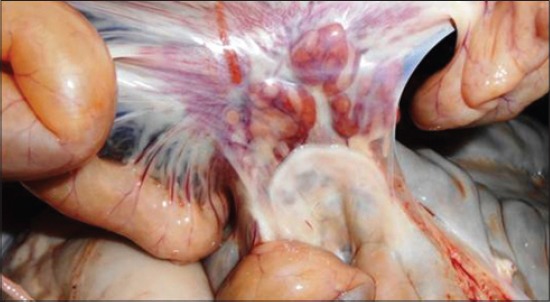
Hemorrhagic lymph nodes on postmortem examination.

Piglets were sacrificed on 11^th^ dpi for post mortem examination. Spleen was enlarged with infarctions of its surface, lymph nodes (mesenteric) were enlarged and hemorrhagic (Figure-3) and petechial hemorrhages on kidneys were observed. Hemorrhagic foci on the intestinal epithelium were also observed. After postmortem examination spleen and lymph nodes were collected and stored at −80°C. CSF challenge virus was found to be highly virulent and produced typical sign and symptoms in the infected piglets. The marked leucopenia also suggests the virulence characteristic of the challenge virus [2,3,5]. On post mortem examination, hemorrhages on the spleen and mesenteric lymph nodes were found, which suggests the acute nature of the disease [2-4]. Histopathological sections of infected spleen were also suggestive of virulent CSFV infection.

### Confirmation of CSFV by reverse transcription PCR (RT-PCR)

**Figure-4:**
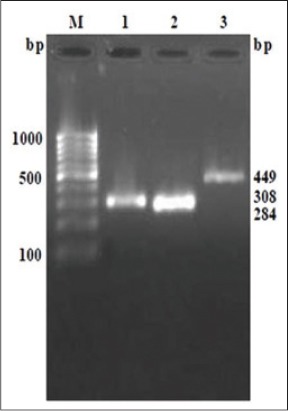
Polymerase chain reaction (PCR) products of 5’ non-translated regions (5`NTR), E2 and NS5B, Lane M: 100 bp marker, Lane 1: PCR product of E2, Lane 2: PCR product of 5`NTR, Lane 3: PCR product of NS5B

On RT-PCR of the respective regions, products size of 284, 308 and 449bp were amplified for 5`NTR, E2 and NS5B respectively as shown in Figure-4. These three regions are importance for molecular diagnostic and genetic typing of the virus isolates [3,4,13].

### Molecular characterization by phylogenetic analysis

**Figure-5:**
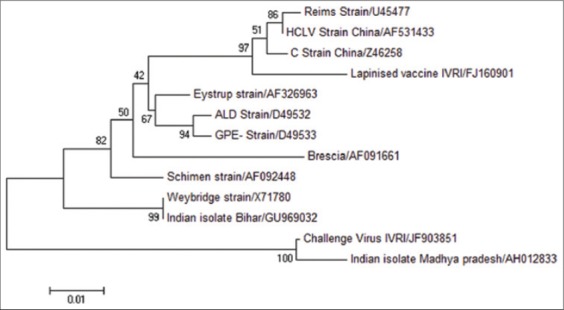
Phylogenetic tree on the basis of 308 bp E2 region

On phylogenetic analysis of 308 bp region of E2 gene CSF challenge virus was clustered with the other virulent viruses and strains (ALD, Eystrup, Brescia, Weybridge, Schimen), and most closely related to the Madhya Pradesh isolate, while most of the vaccine viruses (Reims, HCLV China, C strain China, Lapinized Indian Vaccine) are clustered together in the different clade (Figure-5). Phylogeny of E2 region suggests genetic similarity of CSF challenge virus with some Indian isolates and virulent CSF strains.

**Figure-6:**
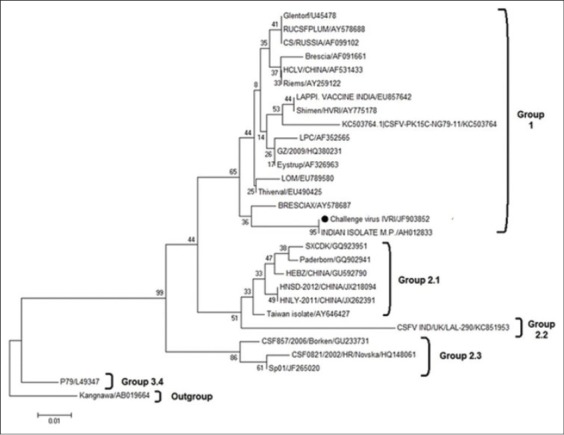
Phylogenetic analysis on the basis of 150 bp region of 5’ non-translated regions

On phylogenetic analysis of 150 bp region of 5`NTR region, virus can be grouped into various groups and subgroups [8,17]. Using the same method CSF challenge virus was grouped under Group 1 along with Indian isolate from Madhya Pradesh and BresciaX strain (Figure-6). 5`NTR being the conserved region of CSFV genome is also being used for genus specific PCR of pestiviuses. Due to conserved nature, 150 bp region of 5`NTR was not able to categorize Group 1 into subgroups, although it can categorize different viruses of other groups into subgroups [8,18].

**Figure-7:**
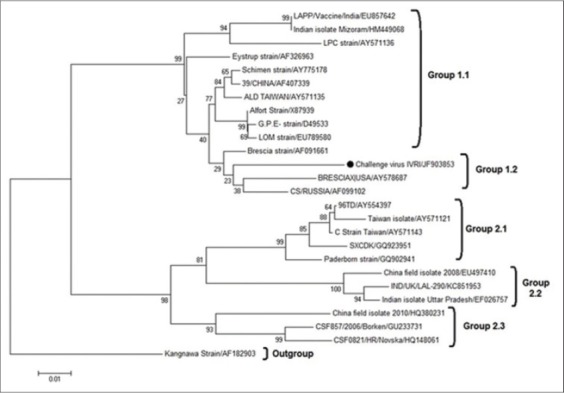
Phylogenetic analysis on the basis of 409 bp region of NS5B

On phylogenetic analysis of 409 bp region of NS5B region, CSF challenge virus was grouped into Group 1 and subgroup 1.2 as already described [8-10]. On phylogenetic comparison of NS5B region also CSF challenge virus was found most closely related to BresciaX strain, followed by Brescia strain of Switzerland (Figure-7). Since Phylogenetic tree corresponding to NS5B region have more boot strap value compared to that of 5`NTR, NS5B based alignment has been found to be more reliable when used in single set [8]. We have also observed the similar finding.

## Conclusion

On the basis of clinical signs, postmortem legion and genetic characterization of 5`NTR, E2 and NS5B regions the CSF challenge virus have been found to be a highly virulent virus belonging to Group 1.2 which is more closely related to Indian field outbreak isolate of Madhya Pradesh and BresciaX strain of USA, followed by Brescia strain of Switzerland. This is the first report of characterization of Indian CSF challenge virus which describes it to be a highly virulent and designated as 1.2 on the basis of partial genome.

## Author’s Contribution

PK, PD implemented the study design. PK and VU carried out the work part and PK, VU and PD did analysis part and wrote the manuscript. All authors have read and approved the final manuscript.
